# Mortality effects of heat waves vary by age and area: a multi-area study in China

**DOI:** 10.1186/s12940-018-0398-6

**Published:** 2018-06-11

**Authors:** Lingyan Zhang, Zhao Zhang, Tao Ye, Maigeng Zhou, Chenzhi Wang, Peng Yin, Bin Hou

**Affiliations:** 1 State Key Laboratory of Earth Surface Processes and Resources Ecology, Faculty of Geographical Science, Beijing Normal University, Beijing, 100875 China; 2 National Center for Chronic and Noncommunicable Disease Control and Prevention, Chinese Center for Disease Control and Prevention, Beijing, 100050 China

**Keywords:** Mortality, Heat wave, Population vulnerability, Probabilistic risk assessment, China

## Abstract

**Background:**

Many studies have reported an increased mortality risk from heat waves comparing with non-heat wave days. However, how much the mortality rate change with the heat intensity―vulnerability curve―is still unknown. Such unknown information makes the related managers impossible to assess scientifically life losses from heat waves, consequently fail in conducting suitable integrated risk management measures.

**Methods:**

We used the heat wave intensity index (HWII) to characterize quantitatively the heat waves, then applied a distributed lag non-linear model to explore the area-specific definition of heat wave, and developed the vulnerability models on the relationships between HWII and mortality by age and by area. Finally, Monte Carlo method was run to assess and compare the event-based probabilistic heat wave risk during the periods of 1971–2015 and 2051–2095.

**Results:**

We found a localized definition of heat wave for each corresponding area based on the minimum AIC (Akaike information criterion). Under the local heat wave events, the expected life loss during 1971–2015 does distinguish across areas, and decreases consistently in the order of WZ Chongqing, PK Nanjing and YX Guangzhou for each age group. More specifically, for the elders (≥65), the average annual loss (AAL) (and 95% confidence interval) would be 61.3 (30.6–91.9), 38 (3.8–72.2) and 18.7 (7.3–30) deaths per million people. With two stresses from warming and aging in future China, the predicted average AAL of the elders under four Representative Carbon Pathways (2.6, 4.5, 6.0, and 8.5) during 2051–2095 would be 2460, 1675, 465 deaths per million for PK Nanjing, YX Guangzhou and WZ Chongqing, respectively, approximately becoming 8~ 90 times of the AAL during 1971–2015.

**Conclusion:**

This study found that the non-linear HWII–mortality relationships vary by age and area. The heat wave mortality losses are closely associated with the social-economic level. With the increasing extreme climatic events and a rapid aging trend in China, our findings can provide guidance for policy-makers to take appropriate regional adaptive measures to reduce health risks in China.

**Electronic supplementary material:**

The online version of this article (10.1186/s12940-018-0398-6) contains supplementary material, which is available to authorized users.

## Background

Interests in health impact of heat wave have being increased with the increasing evidence that extreme climatic events are becoming more frequent, more intense, and longer-lasting [1, 2], and the hazardous effects of heat wave on mortality have been found across various countries [3–7]. Moreover, heat wave can be a big threat in urban area because of the “urban heat island (UHI) effect” that can heat the ambient temperature in urban regions and exacerbate the negative heat effect on residents [8, 9]. Several studies have observed a particularly increased risk of mortality from heat waves on the individuals living in urban environments due to the UHI effect [10, 11]. With the enlarging urban area and the increasing urban population in developing countries, it is expected that populations in cities will carry the heavier burden from heat stress. Therefore, it is highly necessary to deepen our knowledge of public health risk from heat waves, and such study will benefit urban residents to take scientific countermeasures.

Many studies have quantified the temperature–mortality association and found the increased mortality risk from the extreme high temperature [2, 7, 12]. Many others also found an increased mortality risk during heat wave days compared with non-heat wave days [7, 13, 14]. However, such studies had not identified the quantitative relationship between the intensity of heat wave and mortality rate. The relationship is so-called vulnerability curve [15], meaning how much mortality ratio change with intensity of heat wave, which is the key technique and hot issue to assess health risk. Moreover, the vulnerability curve at a local scale can be helpful for government and policy maker to design effective strategies.

To date, the heat-health warning systems are widely used across countries. However, failure in preventing and preparing risk can have tragic consequences — often leading to widespread life losses [16] — such as the 2003 European heat wave [17] and the 2010 Russian heat wave [18], each resulted in more than 70,000 and 11,000 deaths, respectively. Therefore, we should adopt integrated risk management approaches to reduce the life losses from heat waves, which in theory include strategies for disaster prevention, early warning (prediction) systems, disaster mitigation, preparedness and response, and human resource development [16, 19]. In practice, China’s National Development and Reform Commission promulgated the City Climate Action Planning that proposes to construct climate-adapted city, and emphasized that identifying and characterizing public health risks from heat waves in urban areas are important tasks for climate action planning [20]. Overall, estimating mortality loss according to the vulnerability curves could be one of the most important outputs among all risk management strategies.

This study aimed to: (1) based on the area-specific definition of heat wave, identify the quantitative relationships between heat wave intensity and mortality for different age group people; (2) assess a probabilistic heat wave risk for the potential mortality losses among the exposed populations; (3) project and compare the probable mortality losses in the future relative to baseline years. To our knowledge, this is the first quantitative health risk study of heat wave in China, and our findings may provide important information for local governments to develop effective area-specific integrated risk management measures to cope with heat waves.

## Material and methods

### Studied areas

**Fig. 1:**
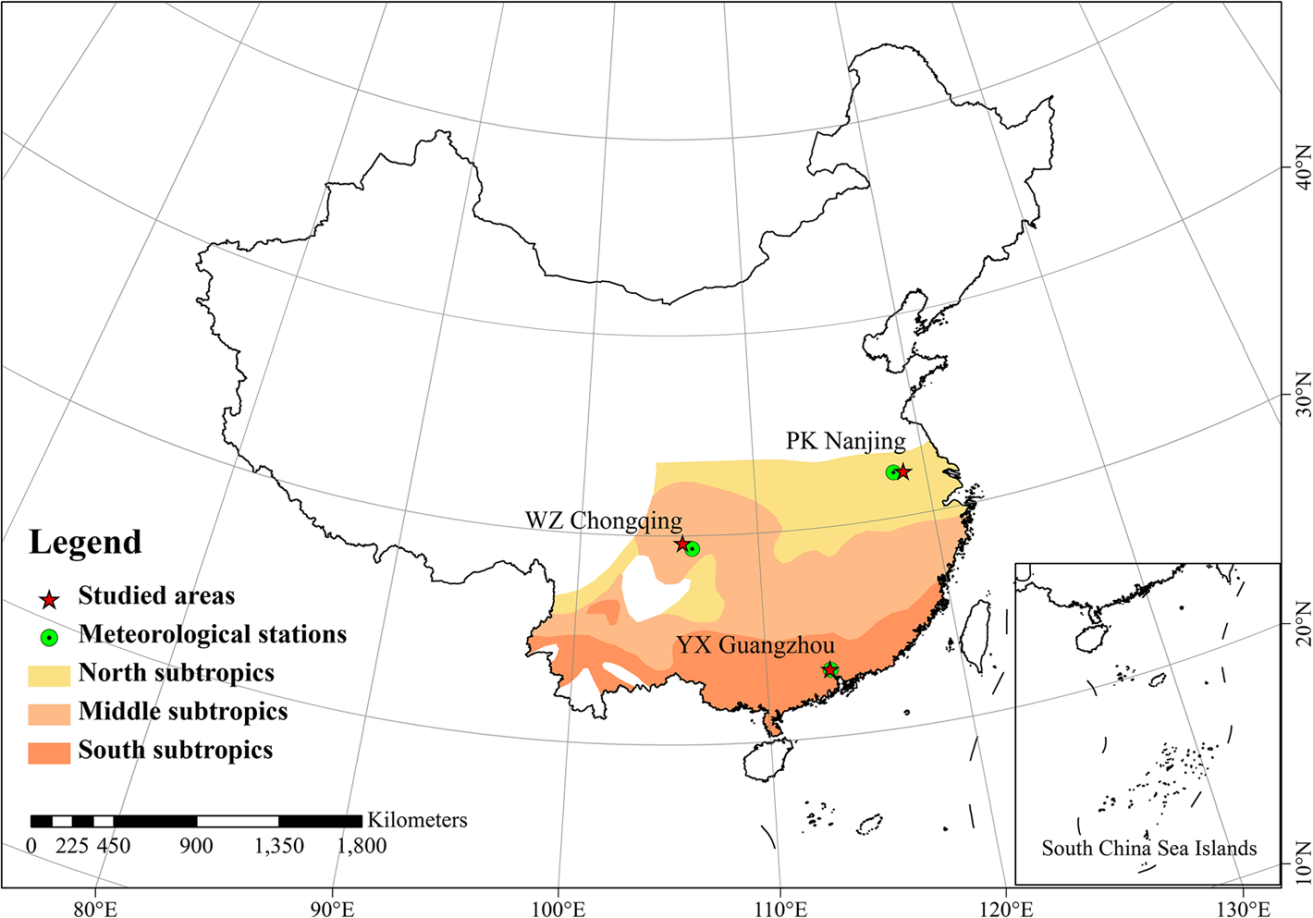
Locations of the studied areas in China

Many previous studies have found that the heat wave effects have the spatial heterogeneity [2, 7, 12] and the urban areas have higher risk partly due to the UHI effect caused by the urbanization [10, 11, 21]. Therefore, to explore the spatial difference of heat wave effects cross China, we selected the studied areas according to following criterions: 1) frequent and intensive occurrence of heat wave; 2) huge differences in spatial location, environment and climatic conditions; 3) being located in a metropolis; 4) available mortality records with high quality. Finally, three districts including PuKou, WanZhou, and YueXiu, which are located in Nanjing, Chongqing and Guangzhou, respectively, were selected. The districts are representative areas located in the north, middle and south subtropics zones of Chinese metropolises, each experiencing hot summers and frequent hot waves (Fig. 1). Particularly, Nanjing and Chongqing are generally known as the Chinese furnace cities. Nanjing is located in China’s eastern coastal area, belongs to the north subtropics with a subtropical monsoon climate and four distinct seasons. Chongqing is located in central China, belongs to middle subtropics with a subtropical monsoon humid climate, mild winter and hot summer. Guangzhou is located in southern China, belongs to south subtropics with subtropical climate, warm winter and hot summer.

From 1971 to 2015, the highest maximum temperature in Nanjing, Chongqing and Guangzhou was 40.4 °C (on August 11, 2013), 43 °C (on August 15, 2006) and 39.1 °C (on July 1, 2004), respectively. Moreover, according to the China City Statistical Yearbook [22], at the end of 2015, the population in the three densely populated cities (Nanjing, Chongqing and Guangzhou) was 6.5 million, 13.9 million and 6.8 million, respectively. In each city, the studied area is district, which is equivalent to the county in Chinese administrative division unit. The PuKou district of Nanjing city will be called as PK Nanjing, the same for WanZhou district of Chongqing city as WZ Chongqing and YueXiu district of Guangzhou city as YX Guangzhou later.

### Data source

The daily non-accidental mortality data of each studied area was observed continuously from 1 January 2007 to 31 December 2012, which was obtained from the Death Surveillance Points System (DSPS) administrated by the Chinese Center for Disease Control and Prevention (China CDC). In the 10th International Classification of Diseases (ICD-10), the non-accidental mortality was explained as deaths not resulting from external causes and coded as A00–R99 [23].

To match mortality records, social economic data during the same period was obtained from China City Statistical Yearbook [22] and included area, permanent resident population size and per capita Gross Domestic Product (GDP). The population distribution records were obtained from the China Health and Family Planning Statistical Yearbook [24] according to the Sixth National Census in 2010. Moreover, we calculated the daily crude non-accidental mortality rate as following formula to conduct a temporal and spatial comparison:

1 $$ {Yr}_i^t={Y}_i^t/{P}_i $$

where ${Yr}_i^t$ refers to the crude non-accidental mortality rate on day *t* of year *i*; ${Y}_i^t$ refers to the non-accidental mortality count on day *t* of year *i*; and *P*_*i*_ refers to the permanent resident population at the end of year *i*.

The daily meteorological data from 1 January 1971 to 31 December 2015 was obtained from the nearest national climate station which is available in the publicly accessible China National Weather Data Sharing System (http://data.cma.cn/). The meteorological variables selected in our study include daily maximum, minimum, and mean temperature (°C) and relative humidity (%).

Additionally, the air pollution data from 1 January 2007 to 31 December 2012 was collected from the Ministry of Environmental Protection of China (http://113.108.142.147:20035/emcpublish). This dataset has been used in many previous studies [25, 26]. Air Pollution Index (API) was used to represent the air pollution condition in China. API depicts the air quality and air pollution level by using concentrations of five main air pollutant (i.e. sulfur dioxide (SO_2_), nitrogen dioxide (NO_2_), carbon monoxide (CO), ozone (O_3_) and particulate matter with particle size below 10 μm (PM10)). The API data was published by China National Environmental Monitoring Centre. Additional file 1: Table S1 shows the association between the level of API and its health impact, as well as the corresponding air pollutant concentrations.

The future climate datasets from 2051 to 2095 (the same studied period as the historical analysis (1971–2015)) are generated by HadGEM2-ES simulation with four representative concentration pathways (RCP) 2.6, 4.5, 6.0, and 8.5 and these data have been bias-corrected to ensure long-term statistical agreement with the observation-based watch forcing data over the period of 1971–2015 [27].

### Definition and quantification of heat wave

Heat wave is one of the extreme weather events associated with climate change, and generally recognized as a ‘prolonged period of excessive heat’ [28]. The definitions of heat wave vary by regions with different geographical location, climatic environment and acclimatization of the local population. Therefore, a local definition of heat wave is needed to better characterize the intensity of heat wave, and further help to identify the quantitative association between heat wave and mortality risk. Based on the different definitions in temperature metric, threshold, and duration, we summarized sixteen heat wave definitions (HWs) that have been found to be significantly associated with daily mortality in different countries to explore the localized heat wave definition [3, 29–33] (see Additional file 1: Table S2).

Moreover, to characterize and quantify heat wave, an index used widely in ecology, is firstly defined and applied in epidemiology [34, 35]. The Heat Wave Intensity Index (HWII) is calculated by the following function:

2 $$ HWII=\sum \limits_{t=1}^{dur}{Tmetric}_t- thr $$

where *t* is the index of heat wave day, *dur* is duration (in days) of the heat wave, *thr* is threshold used in the HW, *Tmetric* is the temperature metric used in the HW.

### DLNM and its key parameters

In epidemiologic field, the distributed lag non-linear model (DLNM) has been widely used to investigate the health effect of extreme heat [7, 36, 37] with the ability of examining the non-linearly bi-dimensional exposure–response associations along the space of predictor and lag [38, 39]. Therefore, the DLNM combined with a linear regression was used to estimate the relationships between HWII and mortality in this study. Firstly, we built a “cross-basis” metric for HWII and lag days, and then included it in the linear regression model after controlling the confounders: daily mean temperature, relative humidity, long-term and seasonal trends, day of the week and holidays. The HWII–mortality relationship was described using the following formula:

3 $$ {\displaystyle \begin{array}{c}{Yr}_t=\alpha + cb\left({HWII}_t, lag\right)+{\beta}_1{Tmean}_t+{\beta}_2{Rh}_t+{\beta}_3{Time}_t\\ {}+{\beta}_4{Dow}_t+{\beta}_5{Holiday}_t\end{array}} $$

where *Yr*_*t*_ refers to the crude mortality rate on day *t*; *α* is the intercept; *cb* means cross-basis function, defined by a natural cubic spline with 3 degrees of freedom (*df*) for the lag space and a natural cubic spline with 2 *df* for the HWII space, centered at minimum-mortality HWII that corresponds to the lowest mortality rate; *HWII*_*t*_ refers to the intensity of heat wave, which equals to the HWII of heat wave when day *t* was a heat wave day while equals to 0 when day *t* was a non-heat wave day; *Tmean*_*t*_ refers to the mean temperature on day *t*; *Rh*_*t*_ refers to the relative humidity on day *t*; *Time*_*t*_ refers to the time variable to adjust for time trends; *Dow*_*t*_ is a categorical variable for day of week; *Holiday*_*t*_ is the binary variable indicating public holidays; and *β*_(1–5)_ are respective coefficients. A maximum lag of 3 days was used because previous studies find that the heat effect usually last for 3 days [40–43], and the *df* in the model were all chosen by the Akaike information criterion (AIC). To better compare the HWII–mortality relationships among different areas, HWII was standardized as the area-specific value expressed by the related area-specific percentiles.

Moreover, sensitivity analyses were conducted by changing the df (3–5) for days of lag, df (2–4) for HWII, and maximum lag (3, 5, 7 and 10 days) in the DLNM. After identifying the above three key parameters for DLNM, we also conduct sensitivity analyses for the maximum temperature, minimum temperature and air pollution index (API) metrics in the models to check the robustness of the estimated HWII–mortality relationships.

### Probabilistic risk assessment

We adopted a probabilistic approach to assess heat wave risk proposed by Jayanthi et al. [44] which was widely accepted for other natural phenomena [19, 45–47]. The risk was expressed in terms of average annual loss (AAL), and the loss exceedance probability (EP) curve that can express the probable maximum losses (PML) for different return periods. The basic framework of the probabilistic risk assessment consists of the following three steps.

#### Step1: Characterizing the heat wave events

In each area, the DLNMs for 16 HWs were firstly analyzed to investigate the local level definition of heat wave that corresponded to the model with the lowest AIC value as the previous studies had suggested [2, 13]. The AIC values of 16 HWs for each area were summarized in Additional file 1: Table S3, and the best fit HW is daily average temperature > 98th percentile (31.2 °C) for over 2 consecutive days, daily maximum temperature > 35 °C for over 3 consecutive days and daily maximum temperature > 95th percentile (35.3 °C) for over 2 consecutive days for PK Nanjing, WZ Chongqing and YX Guangzhou, respectively. Then, heat waves from two periods of 1971–2015 and 2051–2095 were identified based on the localized HW and quantified using HWII. Finally, we fitted the probability distributions for the annul frequency and intensity of HWII for each period.

#### Step2: The exposures and their vulnerability

The exposed elements during heat wave periods were populations, which were grouped by age (0–64 years and over 64 years). The vulnerability curves define the crude mortality rate (CMR) loss among the exposed populations caused by a given heat wave intensity [15], which were estimated as the quantitative relationships of HWII–mortality derived from the DLNMs according to a local-specific definition of heat wave (see section “DLNM and its key parameters” for details).

#### Step3: Event-based heat wave risk assessment

A Monte Carlo simulating stochastic 10,000 years’ heat wave events was performed using the frequency and intensity function obtained in *Step 1*. According to the vulnerability curves obtained in *Step 2*, we estimated the CMR losses for each stochastic heat wave event, as well as the annual CMR losses. To consider the demographic effect and compare the losses across areas more accurately, we convert the CMR losses to age-specific mortality rate (ASMR) losses according to the population age structure of the Sixth National Census in 2010 in each studied area. Based on ASMR losses over years, we further could construct the EP curves. Then, the AAL and PML at given return period (the inverse of the annual probability of exceedance) was calculated. For example, a 1-in-100 year PML is the lower limit on the loss at a 1% probability of exceedance on the EP curve.

As for the future projection, we projected AAL for two age groups during 2051–2095 based on the following two assumptions: (1) Due to the population age structure of different areas in the future unavailable, the same aging trend for each area was pre-assumed. According to the latest report, the number of the elderly aged 65 and over is expected to reach 318 million and representing 31.53% of the total population by 2095 [48]; (2) the vulnerability curves of three areas in the future will keep same as those during 2007–2012, without considering any improvement in accustomed ability or countermeasures to heat wave.

The ‘dlnm’ package in R software version 3.2.5 was used to develop the vulnerability curves. The Matlab statistical software version R2014a was used to perform the Monte Carlo analysis.

## Results

We summarized the information on the daily average mortality counts for different age groups, meteorological condition, vulnerable population and socio-economic condition in each studied area (Table 1). Overall, 89, 002 deaths from 2007 to 2012 were recorded in the study, with YX Guangzhou having the most daily average non-accidental deaths. Mean temperature and relative humidity were annual averages from 2007 to 2012, with YX Guangzhou having the highest average mean temperature and WZ Chongqing having the highest average mean humidity. The proportions of the population aged over 64 exceed 7%, implying that each area has entered an ageing society [49]. In addition, the per capita GDP indicates that the economic condition is highest in YX Guangzhou, followed by PK Nanjing and WZ Chongqing.

**Table 1:** Summary mortality, weather statistics, area, population, and economic data in the studied areas during period of 2007–2012

Studied areas	PK Nanjing	WZ Chongqing	YX Guangzhou
Daily average mortality counts			
Age: 0–64 years^a^	2 (0, 7)	4 (0, 17)	4 (0, 2)
Age: 65– years^a^	7 (0, 20)	9 (0, 33)	15 (3, 41)
Meteorological condition			
Relative humidity (%)^a^	72.2 (19, 100)	76.1 (22, 100)	74.5 (25, 100)
Mean temperature (°C)^a^	16.1 (− 6, 34.2)	17.9 (5, 34.7)	22.3 (5.1, 33.5)
Vulnerable population			
The proportion of population aged 65 and over (%)	7.9	11.4	15.1
Socio-economic condition			
Area (km^2^)	912.3	3457	33.8
Population (10,000)	64.6	173.9	116.5
GDP per capita (CNY)	50,338	27,507	130,869

^a^Mean (min, max)

**Fig. 2:**
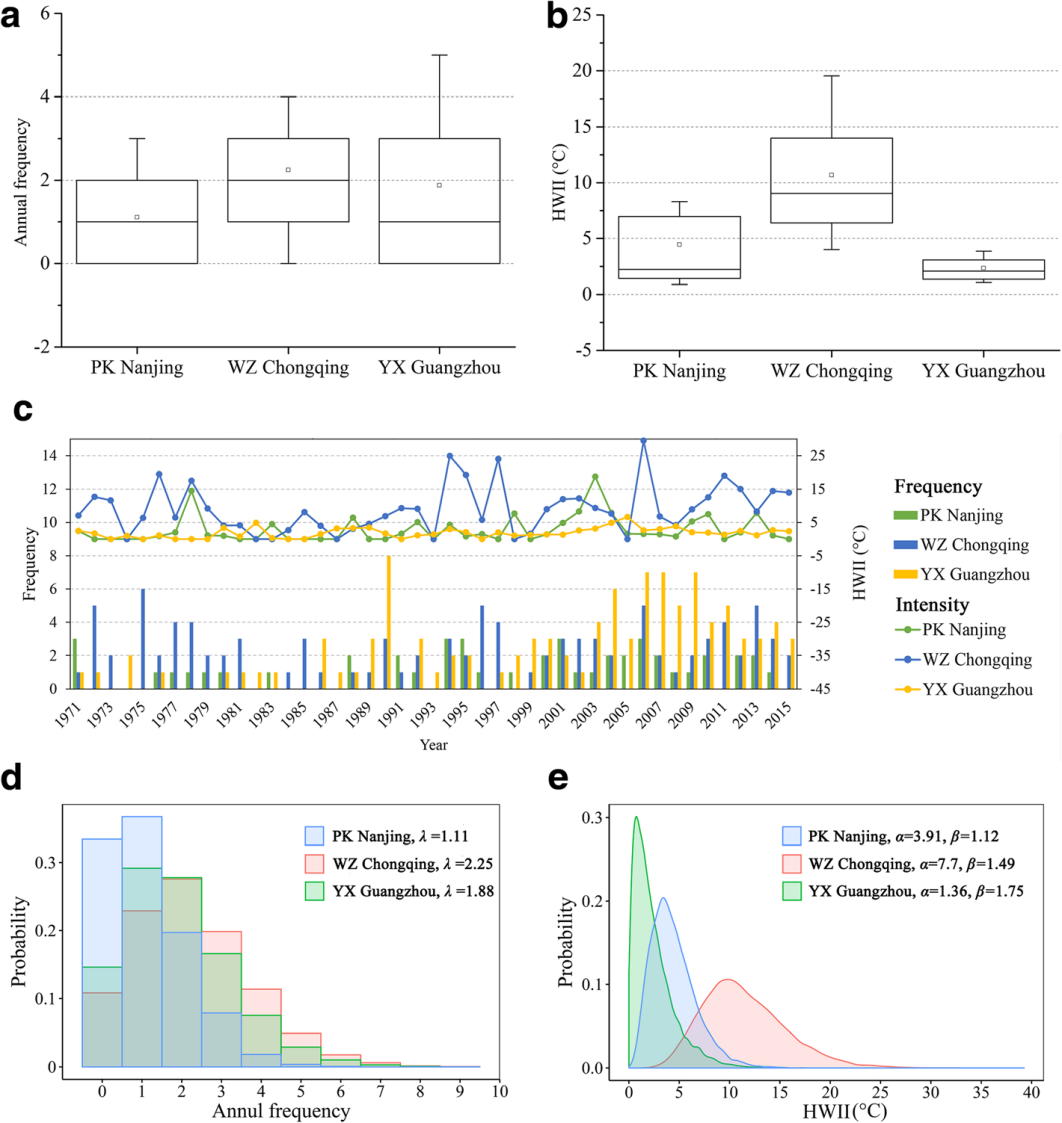
Annual frequency and HWII of heat waves identified from 1971 to 2015 in the studied areas. **a** Box plot for annul frequency of heat waves; **b** Box plot for HWII of heat waves; The boxes present the 25-to-75 percentile, whiskers (i.e., the horizontal dashes linked to boxes by vertical lines) present the 10-to-90 percentile, hollow square and thick dash in boxes are mean and median values. **c** Annual frequency and annual average HWII of heat waves over time; **d** Probability distribution fittings for annul frequency of heat waves, which are Poisson distributions, the *λ* refers to the vector of (non-negative) means. **e** Probability distribution fittings for HWII of heat waves, which are Gamma distributions, the *α* and *β* refer to the shape and scale parameters. All probability distributions have passed the Chi-square test considering a *p* value of 0.01

Figure 2 displays their annual frequency and intensity (HWII) over time with the corresponding box plots and probability distributions. In summary, at least one heat wave event was occurred yearly in all three areas (Fig. 2a), and the average frequency and HWII in WZ Chongqing are the highest (2, 10.7 °C), followed by PK Nanjing (1, 4.4 °C) and YX Guangzhou (2, 2.3 °C) (Fig. 2a-b). Both the annual frequency and HWII of heat waves were fluctuating upward over the past 45 years (Fig. 2c). Moreover, probability distribution fittings for annul frequency of heat waves are Poisson distributions, while they are Gamma distributions for HWII. All distributions have passed the Chi-square test with a *p* value of 0.01. Especially for WZ Chongqing, its frequency and HWII are more right than the others, indicating a higher likelihood of extreme heat waves (Fig. 2d-e).

**Fig. 3:**
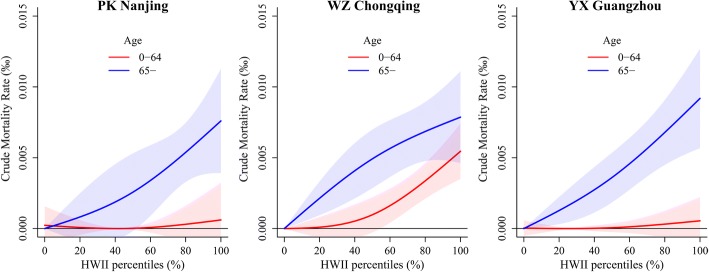
HWII–mortality relationships for different age groups in the studied areas with the lag of 0–3 days, the shaded areas represent 95% confidence intervals

Figure 3 displays the overall quantitative relationships of HWII–mortality (vulnerability curves) for two age groups over 0–3 days, indicating the human vulnerability when exposing to different intensity of heat waves. For the youngers (0–64 years old), the curves rise slightly with HWII increasing in PK Nanjing and YX Guangzhou, while it rises sharply (J-shaped) in WZ Chongqing. For the elders (≥65), the trends of curves rise slowly with a lower HWII (about 40% percentile) and become sharply with higher HWII in PK Nanjing and YX Guangzhou, while it is completely opposite in WZ Chongqing. Overall, the curves for the elders are always above that for the youngers in all studied areas, implying that the elders are more vulnerable to heat wave events than the youngers.

To test the robustness of the vulnerability models, we had also conducted a series of sensitivity analysis. Degrees of freedom (*df*) is a key parameter for DLNM analysis. The sensitive analysis substantiated strongly that the HWII–mortality relationships are very sensitive to the *df* for HWII and *df* for lag days (Additional file 1: Table S4). The best fits with minimum AIC value are consistently indicated at 2 *df* for HWII combined with 3 *df* for lag days in all three studied areas. API was input into DLNM to explore the effect of air pollutants on life loss. By comparing the results without (Fig. 3) and with API (Additional file 1: Figure S1-a), no significant changes have been found for the HWII–mortality relationships. Moreover, the monthly patterns of API showed that the air pollution is much more severe in winter and spring months than summer (Additional file 1: Figure S2). Therefore, the consistently lower API in summer had highlighted that there are very weak associations between heat waves and air pollution in the studied areas, which substantiated strongly no significant added impact of air pollution to the vulnerability models. Apart from the average temperature, we also included the other temperature metrics (maximum and minimum) in the models and found no significant impact to the HWII–mortality relationships (Additional file 1: Figure S1-b, S1-c). With the increasing maximum lag days in the models, although the relationships for the elders changed little, the confidence intervals changed hugely for the youngers (Additional file 1: Figure S3), indicating an aggravated uncertainty. Therefore, the 3 days last of heat effect found in previous study [40–43] and our sensitivity analysis both highlight that the lag days of 0–3 is reasonable in the study.

**Fig. 4:**
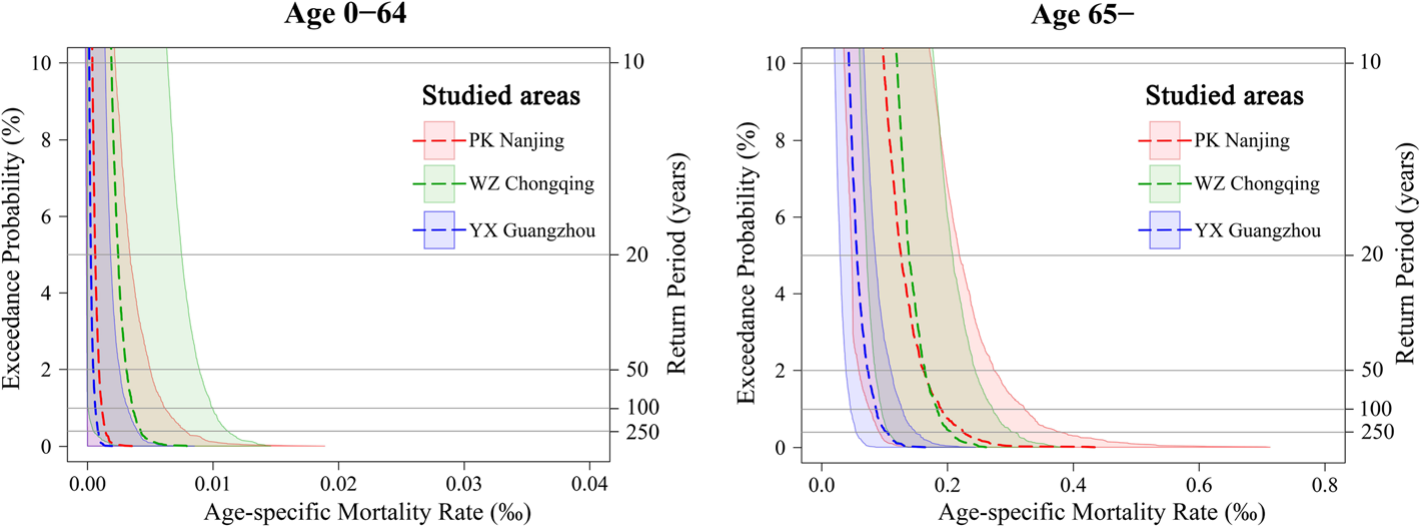
Exceeding probability curves of stochastic 10,000 years’ mortality loss for people 0–64 years old (**a**) and over 64 years old (**b**) in the studied areas during 1971–2015. The shaded areas represent 95% confidence intervals

Figure 4 displays the EP curves of stochastic 10,000 years’ losses with uncertainty (95% confidence interval) for two age groups in three studied areas during baseline years (1971–2015). These EP curves portray life loss in a temporal manner, whose right-hand tail situated the largest loss. The EP curves for the elders are far more right and show a longer right tail than those for the youngers, indicating a higher risk of life loss for people over 64 years old. Overall, for the population aged 0–64, from left to right, the EP curves with increasing uncertainty are, YX Guangzhou, PK Nanjing and WZ Chongqing, respectively. However, when faced server heat waves (return period of more than approximate 50 years), the elder population in PK Nanjing would burden the largest loss among the studied areas.

Table 2 provides an overview of AAL and PML at different given return periods in each studied area during 1971–2015. Generally, the potential death number will multiply with the increasing intensity of heat wave, and the increased times range from 3 to 4 for 1/50a events and 3–5 for 1/100a events, both comparing with AAL. The expected crude heat wave morality loss does distinguish across studied areas, and the consistently decreased order is WZ Chongqing, PK Nanjing and YX Guangzhou for each level of intensity event. However, when take into account of the age structure in each area, the heaviest loss for the elder population was found in PK Nanjing when the server heat waves occur. More specifically, under the heat wave events at return period of 100 years, the decreased order of PML (average, 95% confidence interval) among 1 million residents aged 65 years or over would be PK Nanjing (189.9, 75.9–322.8), WZ Chongqing (183.9, 94.6–271.5) and YX Guangzhou (86, 46–127.3). For the youngers, the PML of that would be WZ Chongqing (3.6, 0–9.9), PK Nanjing (1.1, 0–6.2) and YX Guangzhou (0.6, 0–3.2) among 1 million residents aged 0–64.

**Table 2:** Average annual loss (AAL) and probable maximum loss (PML) at given return period for two age groups during 1971–2015. The numbers in parentheses indicate mortality loss at 95% confidence interval

Age	Studied areas	Heat wave mortality loss (per million)		
		AAL	1/50a	1/100a
0–64^a^	I	0.2 (0, 1.3)	0.9 (0, 5.0)	1.1 (0, 6.2)
	II	0.9 (0, 3.3)	3.0 (0, 8.9)	3.6 (0, 9.9)
	III	0.1 (0, 0.7)	0.5 (0, 2.6)	0.6 (0, 3.2)
65–^a^	I	38.0 (3.8, 72.2)	162.0 (58.2, 273.4)	189.9 (75.9, 322.8)
	II	61.3 (30.6, 91.9)	163.7 (84.9, 244.3)	183.9 (94.6, 271.5)
	III	18.7 (7.3, 30.0)	73.3 (38.7, 110.0)	86.0 (46.0, 127.3)
All^b^	I	3.2 (0.3, 6.9)	13.6 (4.6, 26.2)	16.0 (6.0, 31.2)
	II	7.8 (3.5, 13.4)	21.4 (9.7, 35.8)	24.2 (10.8, 39.8)
	III	2.9 (1.1, 5.1)	11.4 (5.8, 18.7)	13.4 (6.9, 21.8)

I: PK Nanjing; II: WZ Chongqing; III: YX Guangzhou

^a^Age-specific mortality rate

^b^Crude mortality rate

**Fig. 5:**
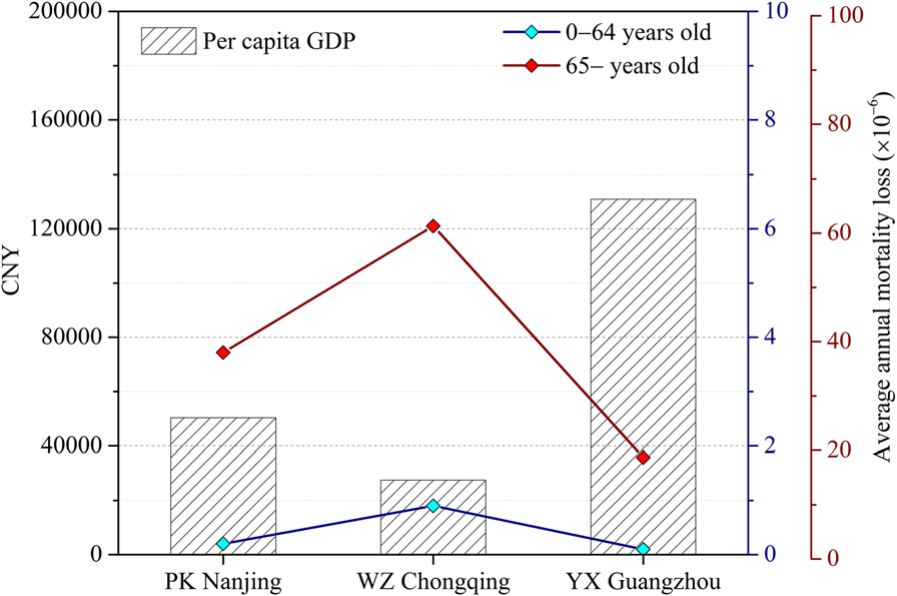
The average annual mortality loss for different age groups and per capita GDP in the studied areas

To discover the potential factors controlling the different risk among areas, we displayed the AAL and the social-economic condition of each studied area (Fig. 5). Per capita GDP is recognized as a widely accepted index to quantify the social-economic condition in a certain area. Interestingly, the change in AAL is totally opposite to Per capita GDP across studied areas, with the highest AAL in the poorest area (WZ Chongqing) while the lowest AAL in the richest area (YX Guangzhou).

In the future, a consistently increase in heat intensity and frequency would be expected under the increased emission scenarios in the three areas (Table 3). The annual averages of intensity and frequency during 2051–2095 and under four RCPs would be 3–8 times of those during 1971–2015. However, very higher increase in mortality losses for the elders would be expected, especially for PK Nanjing and YX Guangzhou, with the AAL values ranging from 1576 to 3861, and 840–3014 per million. The elders in WZ Chongqing would consistently expect the lowest mortality losses, while the highest losses for the youngers in the area under the four scenarios.

**Table 3:** Basic information on heat wave and the projected mortality losses during the period of 2051–2095 under four different scenarios in the studied areas

Scenario	Studied areas	Avereage HWII (°C)	Annual average frequency	Annul Average mortality loss (per million)		
				Age 0–64^a^	Age 65–^a^	All^b^
RCP2.6	I	21.0	6	16.8	1576.1	506.2
	II	26.5	7	26.8	329.7	121.9
	III	10.1	14	10.1	839.9	270.6
RCP4.5	I	21.8	7	18.6	1753.9	563.3
	II	29.7	8	37.7	406.7	153.5
	III	13.2	17	18.6	1382.4	446.7
RCP6.0	I	27.2	8	28.9	2649.8	851.6
	II	33.4	8	45.2	445.8	171.0
	III	14.1	17	20.0	1465.2	473.6
RCP8.5	I	30.2	9	42.8	3861.6	1241.5
	II	44.4	10	89.6	679.3	274.7
	III	23.0	20	45.6	3014.2	977.4
Average	I	25.0	7	26.8	2460.3	790.7
	II	33.5	8	49.8	465.4	180.3
	III	15.1	17	23.6	1675.4	542.1

I: PK Nanjing; II: WZ Chongqing; III: YX Guangzhou

^a^Age-specific mortality rate

^b^Crude mortality rate

## Discussion

In the present study, we firstly depicted vulnerability curves of exposed populations based on localized heat wave definition. Then, we conducted a possibility risk assessment analysis to evaluate the probable heat wave life losses of different periods. To the best of our knowledge, this is the first multi-area study to investigate the quantitative associations of HWII–mortality in different latitudes of China.

To date, many indexes have been proposed and adopted in previous studies to quantify the heat waves, such as the heat wave duration index (HWDI) that measured as the duration days of heat wave [50] and the heat wave magnitude index (HWMI) [51, 52] that defined as the maximum heat wave magnitude for a given year. Moreover, Anderson and Bell measured the average daily mean temperature during the heat wave as the intensity, which assumed the heat waves with same intensity but different duration had the same health impact [3]. Such studies did extend our knowledge of heat wave by quantifying HWs through either duration or magnitude or intensity. However, heat wave is a complex process, their studies had not characterized well heat wave only by considering one aspect. Referring to an index in Ecology, we developed a new index—HWII to characterize heat wave. Due to comprehensively taking both threshold temperature and duration into account, many studies have substantiated the index characterizes well heat waves and can quantify objectively the impact of heat wave on crop production, although the threshold temperature varied by crops and phenological stages [34, 35, 53, 54]. Owing to such referring, the index has successfully used in the study, and highlights a potential application in epidemiology.

Although some previous studies have quantified the mortality risk of heat wave, the relationships were expressed by relative risk, rather than mortality ratio. For example, Anderson and Bell have explored a linear mortality effect of heat wave, indicated by increased relative risk for every 1 °F warming [3]. Many others expressed the relative mortality risk on heat-wave days related to non-heat-wave days [7, 13, 14]. Such studies did help us to identify higher risk areas and higher risk populations but will fail in forecasting the potential life loss when a certain heat wave occurring. Therefore, the vulnerability models in our study provide more detailed information to predict the probable mortality loss from heat waves.

The AALs during 1971–2015 suggest the larger number of death from heat events in WZ Chongqing (Table 2), compared with the other areas. The differences in life loss were strongly associated with the socio-economic factor (Fig. 5). PK Nanjing, YX Guangzhou are both at a relatively higher socio-economic level, with more advanced health care systems and larger improvements in living conditions, all of which may play a significant role for mitigating and adapting heat waves [2, 55]. Moreover, the elders were identified as more vulnerable population, which might be caused by the reducing thermoregulatory capacity over time, taking some medicine that may interfere with the process of heat loss, or even the low health risk perception and fewer adaption measures for the elders to the heat waves [7, 56]. In the future (2051–2095), taking into account the change of age composition, the studied areas would experience between 122 and 1242 mortality losses per million residents per year (Table 3) under the four RCPs resulted from both ageing and warming stresses. Due to distinct differences between vulnerability models of WZ Chongqing and the other two areas, the AAL values for the youngers during 2051–2095 would still be the highest (the same order as those of 1971–2015); while the AAL of WZ Chongqing in the future for the elders would become the lowest (a different order from the historical one) because of the slight increase risk following with the increased HWII values. Therefore, given that the rapid aging trend in China, it is very urgent for policy-makers to design adaptation plans for these vulnerable areas and populations against heat waves.

Estimating probable life losses from heat waves will stimulate policy-makers to develop planning options and tools to cope with heat-wave risk, including allocating the sustained budgetary resources necessary to reduce those potential damages. Moreover, with increasing human health risk under climate change [57] and the better-understood loss implication for morbidity and mortality [3, 7, 58, 59], there is an increase in demand for life/health insurance and risk-management products [60]. The AALs and the EP curves are of particular importance for risk financing schemes or risk transfer instruments [46], which could be the base of further studies in defining strategies for financial protection (e.g. heat wave health insurance) against heat wave losses.

Although we have developed methods to estimate potential life loss from heat wave, there are still some limitations in the study. First, the role of mortality harvesting was not investigated in this study. It is generally known as the fact that short-term mortality increases during a heat event have been observed to be offset somewhat by short-term mortality decreases following the event, and the pattern of it may vary in different locations, populations or even causes of disease [61, 62]. Second, the assumptions — no acclimatization to heat wave and the same vulnerability as the present — are likely oversimplified in the future projection. Sheridan et al. found that acclimatization may reduce heat-related mortality by 37 to 56% from their unacclimatized values in California by 2090s [62]. Third, the results derived in our study will hardly expand to other areas because of the limited studied areas, the localized definitions required for heat waves, and the area-specific vulnerability models. Finally, we also did not address in detail how the HWII–mortality relationships differed by other factors, such as gender, cause of death, the availability of air conditioning, and etc. All issues above are needed to address in further research. Despite these limitations, our study also has notable strengths. First, our study took into account the characteristic of heat wave and the acclimatization of the local population to its local climate to choose the localized definition of heat wave based on the minimum AIC. Second, to the best of our knowledge, this is the first quantitative health risk analysis of heat wave in China, which explored the area-specific vulnerability model. Moreover, this study developed an event-based probabilistic framework to assess the heat wave mortality loss, highlighting a further comprehensive study on heat wave risk management.

## Conclusion

We developed area-specific vulnerability models of heat wave in three urban areas of China, which varied by age and city. The elders are more vulnerable to heat wave events than the youngers, and the mortality losses are closely related to the social economic status. With the increasing extreme climatic events and the rapid aging trend in China in the future, the populations could be more vulnerable and experience a considerable burden from heat wave mortality. The quantitative health analysis and probabilistic risk assessment for heat wave based on the localized heat wave definition in this study can provide guidance for government and private organizations to take regional integrated risk management approaches such as early warning/forecasting system, improvement of the general care for the elder people and heat wave health insurance to reduce health risks in China.

## Additional file

Additional file 1: **Table S1.** Introduction of Chinese Air Pollution Index (API). **Table S2.** Heat wave definitions with different metric, threshold and duration in this study. **Table S3.** Akaike Information Criterion (AIC) values for models examining HWII–mortality relationships over lag 0–3 days by 16 heat wave definitions in the studied areas during 2007–2012. The values in orange are the minimum AICs in each area. **Table S4.** Akaike Information Criterion (AIC) values for models examining HWII–mortality relationships over lag 0–3 days by different degrees of freedom (df) for HWII and days for lag in the studied areas during 2007–2012. The values in orange are the minimum AICs in each area. **Figure S1.** Include API (a), maximum temperature (b) and minimum temperature (c) separately in the HWII–mortality models for different age groups in the studied areas over lag 0–3 days, the shaded areas represent 95% confidence intervals. **Figure S2.** Monthly averages of both API and HWII from 2007–2012 in the studied areas. **Figure S3.** The HWII–mortality relationships for different age groups in the studied areas over lag 0–5 days (a), 0–7 days (b) and 0–10 days (c), the shaded areas represent 95% confidence intervals (DOC 23835 kb).

## Abbreviations

AAL: Average annual loss
AIC: Akaike information criterion
API: Air pollution index
ASMR: Age-specific mortality rate
CMR: Crude mortality rate
DLNM: Distributed lag non-linear model
EP: Exceedance probability
GDP: Gross Domestic Product
HW: Heat wave definition
HWII: Heat wave intensity index
PK Nanjing: PuKou district of Nanjing city
PML: Probable maximum loss
UHI: Urban heat island
WZ Chongqing: WanZhou district of Chongqing city
YX Guangzhou: YueXiu district of Guangzhou city
