# Supplementary material for: Mortality effects of heat waves vary by age and area: a multi-area study in China
Source: Environ Health. 2018 Jun 11;17:54. doi: 10.1186/s12940-018-0398-6 (PMC5996527; doi:10.1186/s12940-018-0398-6)
Supplement: Supplementary file 1 — Table S1. Introduction of Chinese Air Pollution Index (API). Table S2. Heat wave definitions with different metric, threshold and duration in this study. Table S3. Akaike Information Criterion (AIC) values for models examining HWII–mortality relationships over lag 0–3 days by 16 heat wave definitions in the studied areas during 2007–2012. The values in orange are the minimum AICs in each area. Table S4. Akaike Information Criterion (AIC) values for models examining HWII–mortality relationships over lag 0–3 days by different degrees of freedom (df) for HWII and days for lag in the studied areas during 2007–2012. The values in orange are the minimum AICs in each area. Figure S1. Include API (a), maximum temperature (b) and minimum temperature (c) separately in the HWII–mortality models for different age groups in the studied areas over lag 0–3 days, the shaded areas represent 95% confidence intervals. Figure S2. Monthly averages of both API and HWII from 2007–2012 in the studied areas. Figure S3. The HWII–mortality relationships for different age groups in the studied areas over lag 0–5 days (a), 0–7 days (b) and 0–10 days (c), the shaded areas represent 95% confidence intervals (DOC 23835 kb). [file 12940_2018_398_MOESM1_ESM.doc]

# Additional file 1

**Table S1.** Introduction of Chinese Air Pollution Index (API)

**1-1** Air pollution sub-index levels and their corresponding air pollutant concentrations

| **Air pollution sub-index** | **Air pollutant concentrations (μg/m^3^ )** | | | | |
| --- | --- | --- | --- | --- | --- |
|  | SO^2^ 24-h | NO^2^ 24-h | PM_10_ 24-h | CO 24-h | O^3^ 8-h |
| **50** | 50 | 80 | 50 | 5000 | 120 |
| **100** | 150 | 120 | 150 | 10000 | 200 |
| **200** | 800 | 280 | 350 | 60000 | 400 |
| **300** | 1600 | 565 | 420 | 90000 | 800 |
| **400** | 2100 | 750 | 500 | 120000 | 1000 |
| **500** | 2620 | 940 | 600 | 150000 | 1200 |

**1-2 API and health implications (daily targets)**

| **API** | **Air pollution level** | **Health implications** |
| --- | --- | --- |
| **0–50** | Excellent | No health implications |
| **51–100** | Good | No health implications |
| **101–200** | Lightly polluted | Slight irritations may occur, individuals with breathing or heart problems ought to reduce outdoor exercise |
| **201–300** | Moderately polluted | Healthy people will be affected significantly. People with breathing or heart problems will experience reduced endurance in activities. These individuals and elders should remain indoors and restrict activities |
| **300+** | Severely polluted | Healthy people will experience reduced endurance in activities. There may be strong irritations and symptoms and may trigger other illnesses. Elders and the sick should remain indoors and avoid exercise. Healthy individuals should avoid outdoor activities. |

**Table S2** Heat wave definitions with different metric, threshold and duration in this study.

| HW | Definition | Reference |
| --- | --- | --- |
| HW01 | Daily average temperature >90^th^ percentile for ≥2 consecutive days | Anderson and Bell (2011) |
| HW02 | Daily average temperature >95^th^ percentile for ≥2 consecutive days | Anderson and Bell (2011) |
| HW03 | Daily average temperature >98^th^ percentile for ≥2 consecutive days | Anderson and Bell (2011) |
| HW04 | Daily average temperature >99^th^ percentile for ≥2 consecutive days | Anderson and Bell (2011) |
| HW05 | Daily average temperature >90^th^ percentile for ≥3 consecutive days | Son et al. (2012) |
| HW06 | Daily average temperature >95^th^ percentile for ≥3 consecutive days | Son et al. (2012) |
| HW07 | Daily average temperature >98^th^ percentile for ≥3 consecutive days | Son et al. (2012) |
| HW08 | Daily average temperature >99^th^ percentile for ≥3 consecutive days | Son et al. (2012) |
| HW09 | Daily average temperature >90^th^ percentile for ≥4 consecutive days | Tian et al. (2013) |
| HW10 | Daily average temperature >95^th^ percentile for ≥4 consecutive days | Tian et al. (2013) |
| HW11 | Daily average temperature >98^th^ percentile for ≥4 consecutive days | Tian et al. (2013) |
| HW12 | Daily average temperature >99^th^ percentile for ≥4 consecutive days | Tian et al. (2013) |
| HW13 | Daily maximum temperature >95^th^ percentile for ≥2 consecutive days | Anderson and Bell (2011) |
| HW14 | Daily maximum temperature >98^th^ percentile for ≥2 consecutive days | Saha et al. (2015) |
| HW15 | Daily maximum temperature >35°C for ≥3 consecutive days | Zhang et al. (2016) |
| HW16 | Daily maximum temperature >35°C for ≥7 consecutive days and daily average temperature >97^th^ percentile | Ma et al. (2011) |

**Table S3.** Akaike Information Criterion (AIC) values for models examining HWII–mortality relationships over lag 0–3 days by 16 heat wave definitions in the studied areas during 2007–2012. The values in orange are the minimum AICs in each area.

| HW | PK Nanjing | WZ Chongqing | YX Guangzhou |
| --- | --- | --- | --- |
| HW01 | −20080.8 | −17703 | −20606 |
| HW02 | −20080.6 | −17705.6 | −20611.3 |
| HW03 | −20081.7 | −17696 | −20613.2 |
| HW04 | −20079.5 | −17688.9 | −20614.9 |
| HW05 | −20080.7 | −17701 | −20606.2 |
| HW06 | −20080 | −17705.8 | −20610.2 |
| HW07 | −20080.4 | −17694.7 | −20612.7 |
| HW08 | −20074.3 | −17688.3 | −20614.1 |
| HW09 | −20078.9 | −17701.1 | −20605.4 |
| HW10 | −20078.3 | −17706.2 | −20611.1 |
| HW11 | −20074.9 | −17693.5 | −20612.3 |
| HW12 | −20074.3 | −17689.1 | −20614.1 |
| HW13 | −20075.9 | −17708 | −20624.7 |
| HW14 | −20077.5 | −17701.9 | −20614.5 |
| HW15 | −20074.3 | −17712.3 | −20622 |
| HW16 | −20065.3 | −17683.5 | −20597.4 |

**Table S4**. Akaike Information Criterion (AIC) values for models examining HWII–mortality relationships over lag 0–3 days by different degrees of freedom (*df*) for HWII and days for lag in the studied areas during 2007–2012. The values in orange are the minimum AICs in each area.

| Studied areas | *df* for HWII | *df* for days of lag | | |
| --- | --- | --- | --- | --- |
|  |  | 3 | 4 | 5 |
| PK Nanjing | 2 | -20087.1 | -20080.7 | -20080.7 |
|  | 3 | -20083.6 | -20085.2 | -20085.2 |
|  | 4 | -20081.5 | -20078.4 | -20078.4 |
| WZ Chongqing | 2 | -17712.3 | -17708.4 | -17708.4 |
|  | 3 | -17709.3 | -17703.9 | -17703.9 |
|  | 4 | -17705.6 | -17702.7 | -17702.7 |
| YX Guangzhou | 2 | -20624.7 | -20623 | -20623 |
|  | 3 | -20619.1 | -20615.5 | -20615.5 |
|  | 4 | -20614.3 | -20608.7 | -20608.7 |





**Fig**. **S1**. Include API (a), maximum temperature (b) and minimum temperature (c) separately in the HWII–mortality models for different age groups in the studied areas over lag 0–3 days, the shaded areas represent 95% confidence intervals.


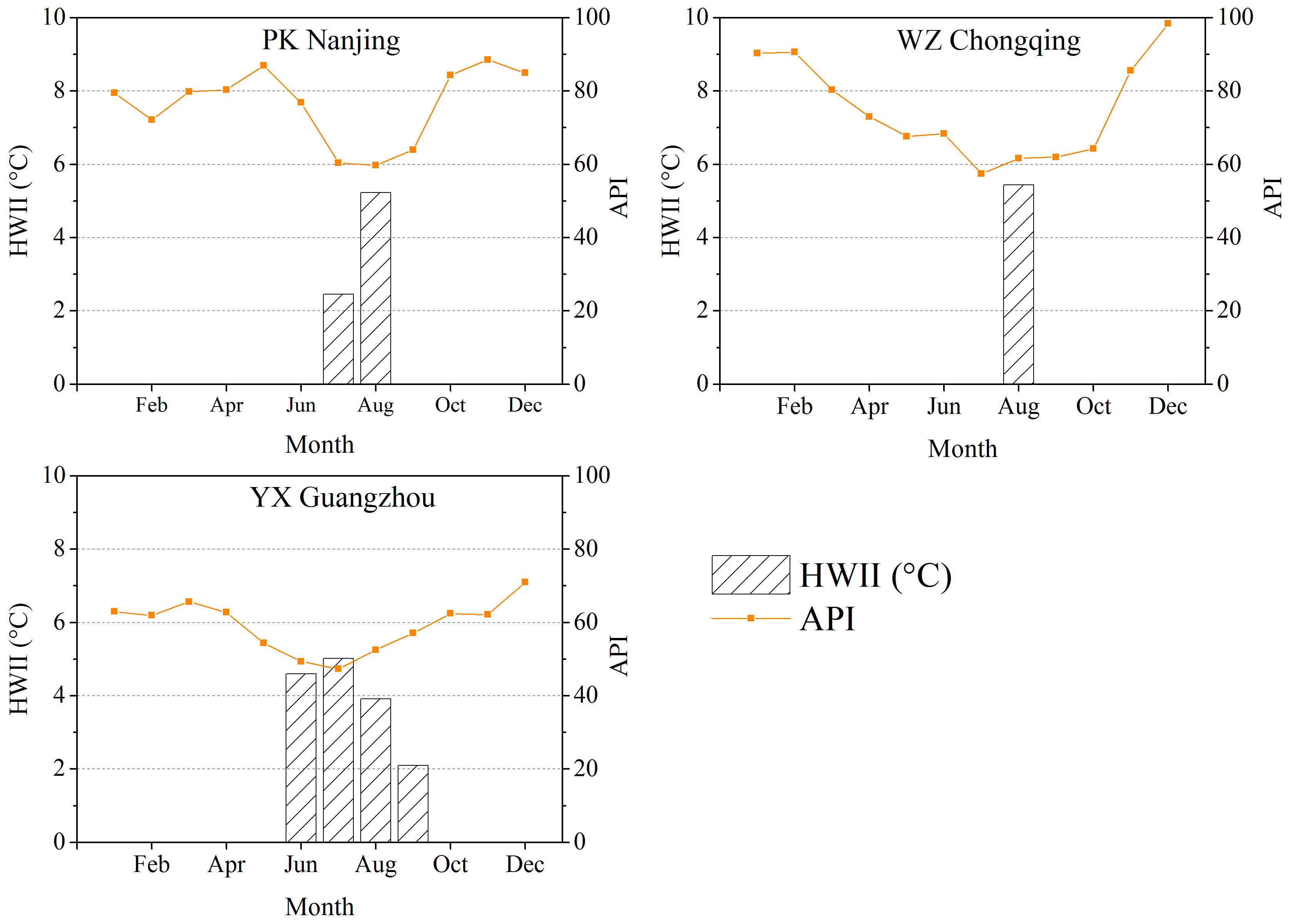


**Fig. S2**. Monthly averages of both API and HWII from 2007–2012 in the studied areas.





**Fig**. **S3**. The HWII–mortality relationships for different age groups in the studied areas over lag 0–5 days (a), 0–7 days (b) and 0–10 days (c), the shaded areas represent 95% confidence intervals.

**References**

Anderson GB, Bell ML. Heat waves in the United States: mortality risk during heat waves and effect modification by heat wave characteristics in 43 U.S. communities. Environ Health Persp. 2011;119(2):210–8.

Ma W, Xu X, Peng L, Kan H. Impact of extreme temperature on hospital admission in Shanghai, China. Sci Total Environ 2011;409:3634–7.

Zhang J, Liu S, Han J, Zhou L, Liu Y, Yang L, Zhang J, Zhang Y. Impact of heat waves on nonaccidental deaths in Jinan, China, and associated risk factors. Int J Biometeorol. 2016;60(9):1367–75.

Son J, Lee J, Anderson BG, Bell ML. The impact of heat waves on mortality in seven major cities in Korea. Environ. Health Persp. 2012;120(4):566–71.

Tian Z, Li S, Zhang J, Guo Y. The characteristic of heat wave effects on coronary heart disease mortality in Beijing, China: a time series study. Plos One. 2013;8(9):e77321.

Saha S, Brock JW, Vaidyanathan A, Easterling DR, Luber G. Spatial variation in hyperthermia emergency department visits among those with employer-based insurance in the United States –– a case-crossover analysis. Environ Health. 2015;14:20.
